# Anticancer effect of terpenes: focus on malignant melanoma

**DOI:** 10.1007/s43440-023-00512-1

**Published:** 2023-07-29

**Authors:** Paula Wróblewska-Łuczka, Justyna Cabaj, Julia Bargieł, Jarogniew J. Łuszczki

**Affiliations:** Department of Occupational Medicine, Medical University of Lublin, Jaczewskiego 8B, 20-090 Lublin, Poland

**Keywords:** Terpenes, Melanoma, Anticancer therapy

## Abstract

Melanoma is a highly aggressive and life-threatening form of skin cancer that accounts for a significant proportion of cancer-related deaths worldwide. Although conventional cancer therapies, such as surgical excision, chemotherapy, and radiation, have been used to treat malignant melanoma, their efficacy is often limited due to the development of resistance and adverse side effects. Therefore, there is a growing interest in developing alternative treatment options for melanoma that are more effective and less toxic. Terpenes, a diverse group of naturally occurring compounds of plant origin, have emerged as potential anticancer agents due to their ability to inhibit tumor growth and induce apoptosis in cancer cells. In this review, the current understanding of the anticancer effects of terpenes (including, thymoquinone, β-elemene, carvacrol, limonene, α-pinene, β-caryophyllene, perillyl alcohol, taxol, betulinic acid, α-bisabolol, ursolic acid, linalool, lupeol, and artesunate) was summarized, with a special focus on their potential as therapeutic agents for malignant melanoma.

## Introduction

**Fig. 1:**
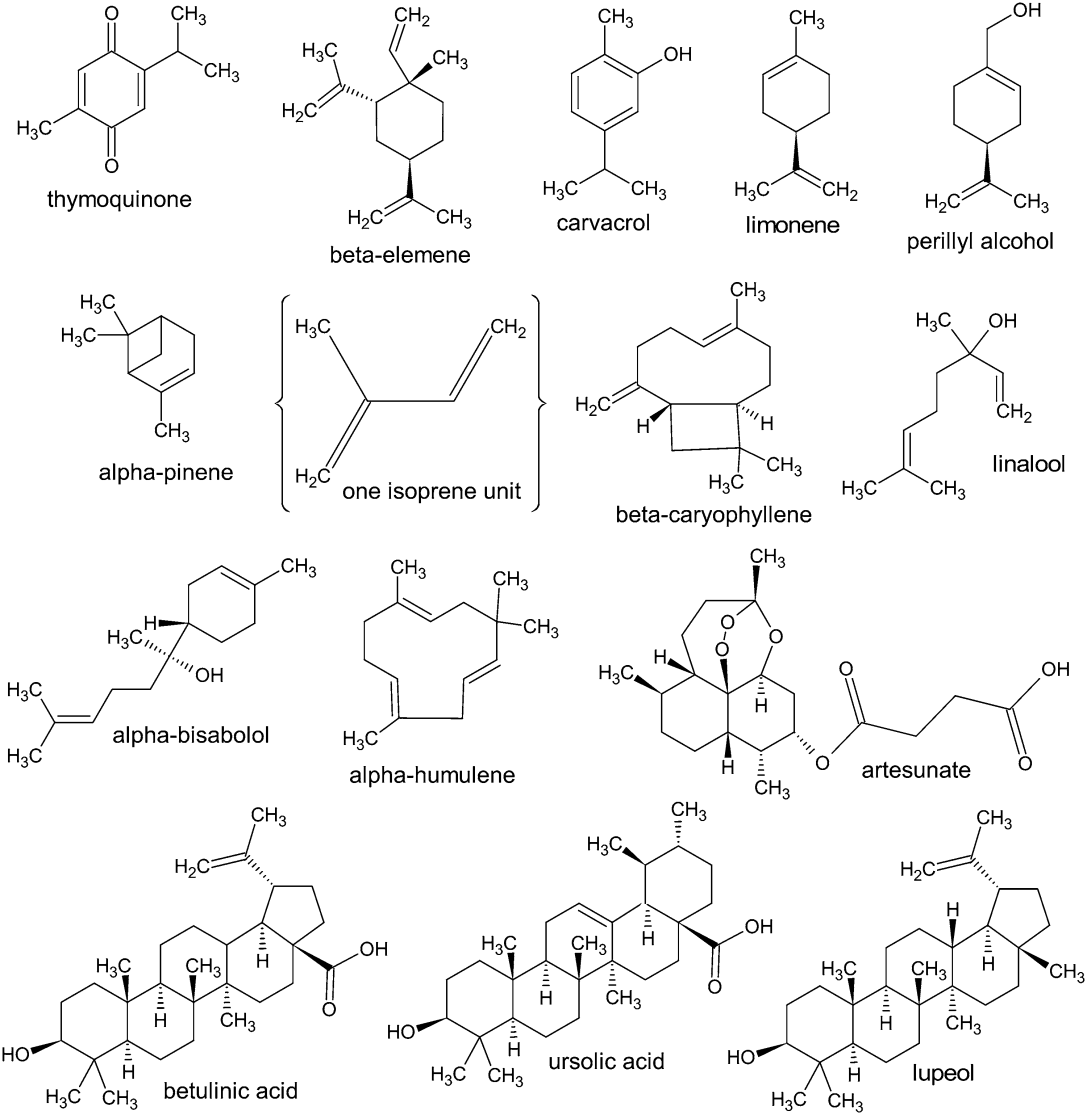
Structural formulas of selected naturally occurring terpenes (ACD/ChemSketch vers. 2021.2.1 software)

Terpenes (also known as isoprenoids) are a diverse large class of organic compounds found in plants, fungi, and some animals [1], characterized by a specific carbon skeleton composed of multiple isoprene units (Fig. 1), which can be arranged in a linear, branched, or cyclic manner [2].

Terpenes play important roles in the biosynthesis of plant secondary metabolites, such as essential oils and pigments, and are involved in various physiological processes, including growth and development, reproduction, and defense against biotic and abiotic stress [3, 4]. Terpenes are synthesized by plants and other organisms through the mevalonate pathway and are often found in essential oils, resins, and other plant-derived materials [5]. Terpenes are the subject of biochemical and molecular research due to their numerous biological activities, including, anticancer, anti-inflammatory, antimicrobial, and antiviral effects [6, 7]. Terpenes interact with specific biological targets, such as enzymes, receptors, ion channels, and can also modulate signaling pathways involved in various cellular processes, including apoptosis, proliferation, and cell differentiation [8].

One of the most notable biological activities of terpenes is their anti-inflammatory effect. Many terpenes modulate the immune system and reduce inflammation by inhibiting the activity of various enzymes and signaling pathways involved in the inflammatory response [9]. Some terpenes possess antioxidant activity, which can protect cells from damage caused by free radicals and oxidative stress [10].

Terpenes have also been reported to exhibit antimicrobial activity against a wide range of bacteria, fungi, and viruses [11]. This effect is thought to be due to the ability of some terpenes to disrupt the cell membrane of microorganisms, leading to their death.

The anticancer properties of terpenes have been widely studied in recent years [12]. Several preclinical studies have demonstrated the potential of terpenes as anticancer agents against various types of cancer, including melanoma [13]. In particular, the use of terpenes as adjuvant therapy in melanoma treatment has gained attention due to their ability to sensitize cancer cells to chemotherapeutic agents and reduce their toxicity [14]. This study aimed to present our expanding knowledge about the mechanisms of action of some terpenes involved in their anticancer effects on melanoma cells.

Melanoma is a type of skin cancer that originates in melanocytes, which are pigment-producing cells located in the basal layer of the epidermis [15]. It is the most aggressive form of skin cancer, with a high potential for metastasis and a poor prognosis if not detected and treated in its early stages [16]. Melanoma accounts for only 1% of all skin cancers, but it is responsible for the majority of skin cancer-related deaths [17].

Melanoma is caused by the accumulation of genetic mutations that disrupt the normal function of melanocytes and promote their uncontrolled proliferation [18]. Exposure to ultraviolet radiation from the sun or artificial sources, such as tanning beds, is the primary environmental risk factor for melanoma [19]. Other risk factors include fair skin, a history of sunburns, a family or personal history of melanoma, and certain genetic mutations [20].

The clinical presentation of melanoma varies depending on the location, stage, and subtype of the tumor. The most common presentation is a pigmented lesion on the skin that changes in size, shape, or color over time [21]. Other signs and symptoms may include itching, bleeding, or ulceration of the lesion, or the appearance of new moles or skin lesions [22].

Melanoma is a particularly challenging type of cancer to treat due to its propensity to metastasize, leading to poor survival rates for patients with advanced disease [23]. Treatment options for melanoma depend on the stage and location of the tumor, as well as the patient's overall health and preferences. Surgery is the primary treatment option for early-stage melanoma, while more advanced cases may require additional therapies, such as chemotherapy, radiation therapy, immunotherapy, or targeted therapy, especially, if melanoma metastases occur [24]. In recent years, there has been a growing interest in the use of natural products, such as terpenes, as adjuvant therapy for melanoma treatment, due to their potential to enhance the efficacy and reduce the toxicity of conventional pharmacotherapy [25, 26]. Additionally, some terpenes inhibit the growth of melanoma cells both in vitro and in vivo [27, 28].

One of the mechanisms by which terpenes may exert their anticancer effects is based on the ability of terpenes to modulate various signaling pathways involved in cell proliferation, apoptosis, and angiogenesis. Another potential mechanism by which terpenes may exhibit their anticancer effects is based on the induction of oxidative stress and DNA damage in cancer cells [29]. This effect can lead to the activation of the cell's apoptosis machinery, resulting in the death of cancer cells.

## Terpenes and cancer: preclinical studies

Preclinical studies have shown that terpenes can induce apoptosis, inhibit cell proliferation, and suppress tumor growth in animal models [30]. For example, β-elemene (a terpene found in plants such as *Curcuma wenyujin*) induces apoptosis in melanoma cells in vitro and inhibits tumor growth in melanoma-bearing mice in vivo [31]. Similarly, carvacrol (a monoterpenoid phenol found in oregano and thyme) inhibits the growth of melanoma cells in vitro and in vivo by inducing cell cycle arrest and apoptosis [32]. Limonene (a monocyclic monoterpene in citrus fruits) inhibits tumor growth and induces apoptosis in melanoma cells in both, in vitro and in vivo studies [33], while α-pinene (a terpene found in pine trees) induces apoptosis and inhibits cell proliferation in melanoma cells in vitro [34].

In addition to their direct anticancer effects, terpenes enhance the efficacy of conventional chemotherapy in preclinical models. For example, β-caryophyllene (a terpene found in many essential oils) sensitizes melanoma cells to doxorubicin by inhibiting the drug efflux pump responsible for drug resistance [35].

Preclinical in vitro studies have demonstrated that various terpenes, such as limonene, β-caryophyllene, and perillyl alcohol, can induce apoptosis, inhibit proliferation, and sensitize melanoma cells to conventional chemotherapy [12, 36]. Moreover, several terpenes have been found to exhibit anti-inflammatory effects, which can also contribute to their anticancer activity. For example, α-pinene inhibits the production of pro-inflammatory cytokines, such as TNF-α (Tumor Necrosis Factor α) and IL-6 (Interleukin 6), in melanoma cells [37]. In addition, β-elemene sensitizes melanoma cells to radiation by inhibiting the DNA damage repair pathway and inducing apoptosis [38]. Limonene enhances radiation-induced DNA damage and cell death in melanoma cells [39], and sensitizes vemurafenib-resistant melanoma cells to the drug by downregulating the expression of the drug efflux pump ABCB1 [40]. Similarly, perillyl alcohol overcomes resistance to BRAF and MEK inhibitors in melanoma cells by inducing apoptosis and inhibiting the MAPK signaling pathway [41].

Accumulating evidence indicates that terpenes exert their anticancer effects not only on melanoma, but also on various human cancers. For instance, taxol (paclitaxel—a diterpene) exerts its anticancer effect by binding to microtubules, stabilizing them, and inhibiting their depolymerization, leading to cell cycle arrest and apoptosis [42]. Thymoquinone (a monoterpenoid found in the seeds of *Nigella sativa* (black seed)), limonene, carvacrol, betulinic acid (a triterpenoid found in the bark of various trees) and α-bisabolol (a sesquiterpene alcohol found in chamomile) induce apoptosis, inhibit cell proliferation, and suppress angiogenesis in various human cancers [43–52].

## Mechanisms of action of terpenes in cancer cells

**Fig. 2:**
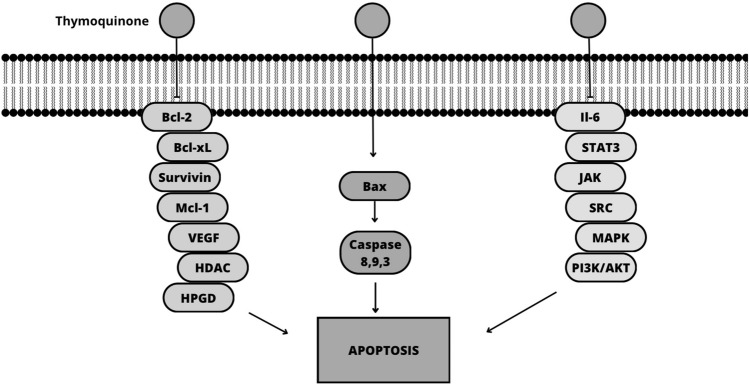
Schematic mechanisms involved in the anticancer effect of thymoquinone on melanoma cells (Canva for Windows)

**Fig. 3:**
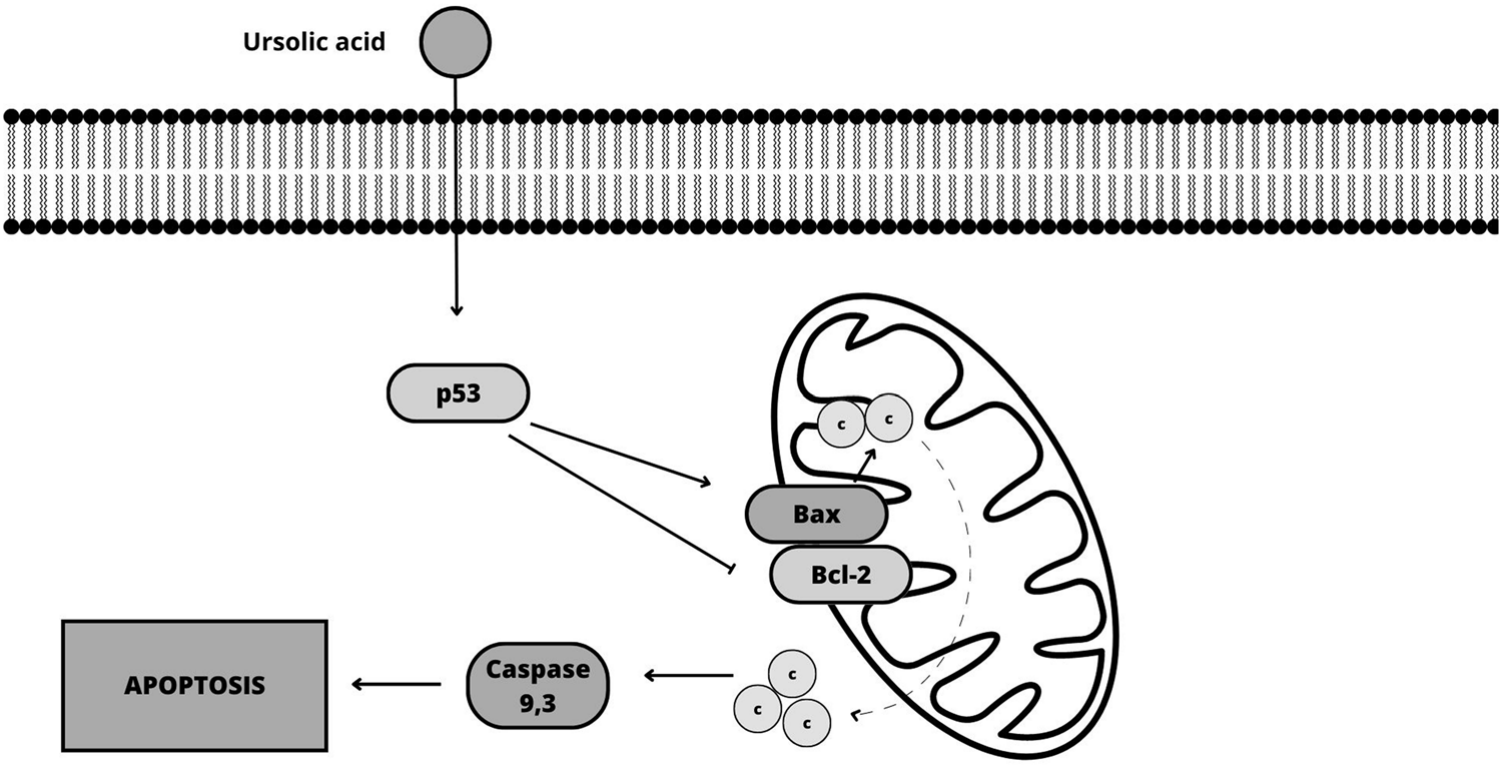
Schematic mechanisms involved in the anticancer effect of ursolic acid on melanoma cells (Canva for Windows)

Accumulating evidence suggests that terpenes exert their effects through multiple molecular mechanisms, including regulation of apoptosis, induction of autophagy, inhibition of cellular signaling pathways, modulation of gene expression, inhibition of angiogenesis, and modulation of inflammation [53, 54]. More specifically, terpenes inhibit the expression of anti-apoptotic proteins, such as Bcl-2 and Bcl-xL, while upregulating pro-apoptotic proteins, such as Bax and caspases, which finally leads to the activation of the apoptotic pathway, promoting cell death [55–58]. Thymoquinone has shown effective results in treating melanoma (MDA-MB-435) by activating the intrinsic apoptosis pathway, while suppressing Akt phosphorylation, and increasing the Bax/Bcl-2 ratio (Fig. 2). This mechanism contributes to the inhibition of cancer cell growth. The presence of highly expressed caspase 3 is associated with the inhibitory effect observed. In addition, in silico target determination has indicated that thymoquinone induces DNA damage by specifically targeting histone deacetylase activity (HDAC) and human 15-hydroxyprostaglandin dehydrogenase (HPGD) [59, 60]. Betulinic acid activates the intrinsic apoptotic pathway and downregulates anti-apoptotic proteins [61]. α-Bisabolol activates the extrinsic apoptotic pathway [62]. The mechanism of action of ursolic acid in B16F-10 melanoma cells involves its inhibitory effect on cell growth by upregulating the expression of p53, Bax, and p21 proteins (Fig. 3). This upregulation leads to the activation of caspase 3-dependent apoptosis, which ultimately results in the programmed cell death of melanoma cells [63].

Terpenes can induce autophagy, a cellular process that helps degrade damaged proteins and organelles, leading to the inhibition of tumor growth [64].

Terpenes inhibit crucial signaling pathways involved in cancer cell proliferation and survival. One of these is the PI3K/Akt/mTOR pathway, which plays a significant role in cell proliferation and survival. Inhibition of this pathway by terpenes leads to apoptosis induction and inhibition of cell proliferation [47, 65]. Terpenes also inhibit the MAPK/ERK signaling pathway, which regulates proliferation, survival, and cell differentiation [66].

**Fig. 4:**
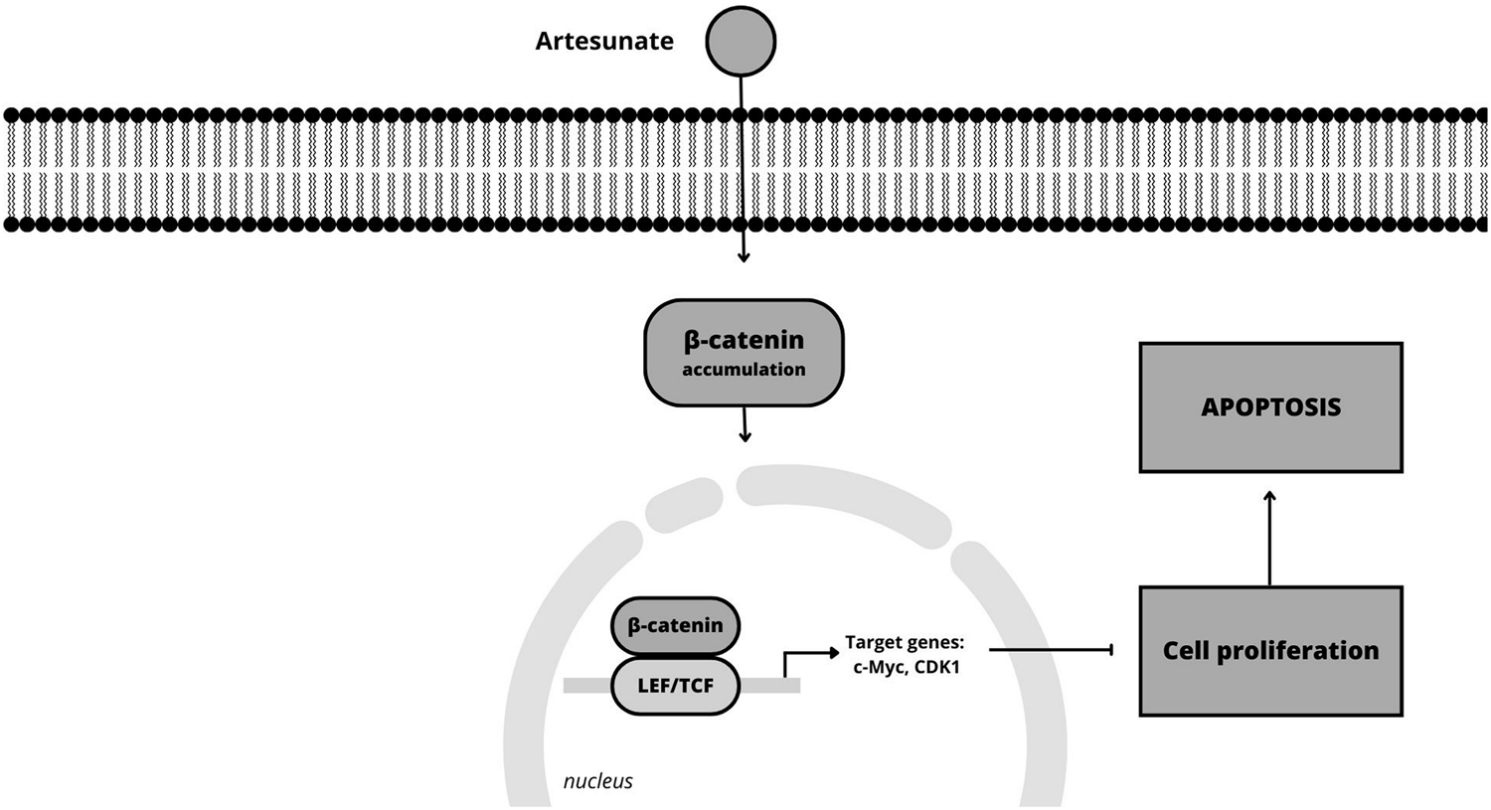
Schematic mechanisms involved in the effect of artesunate on melanoma cells (Canva for Windows)

**Fig. 5:**
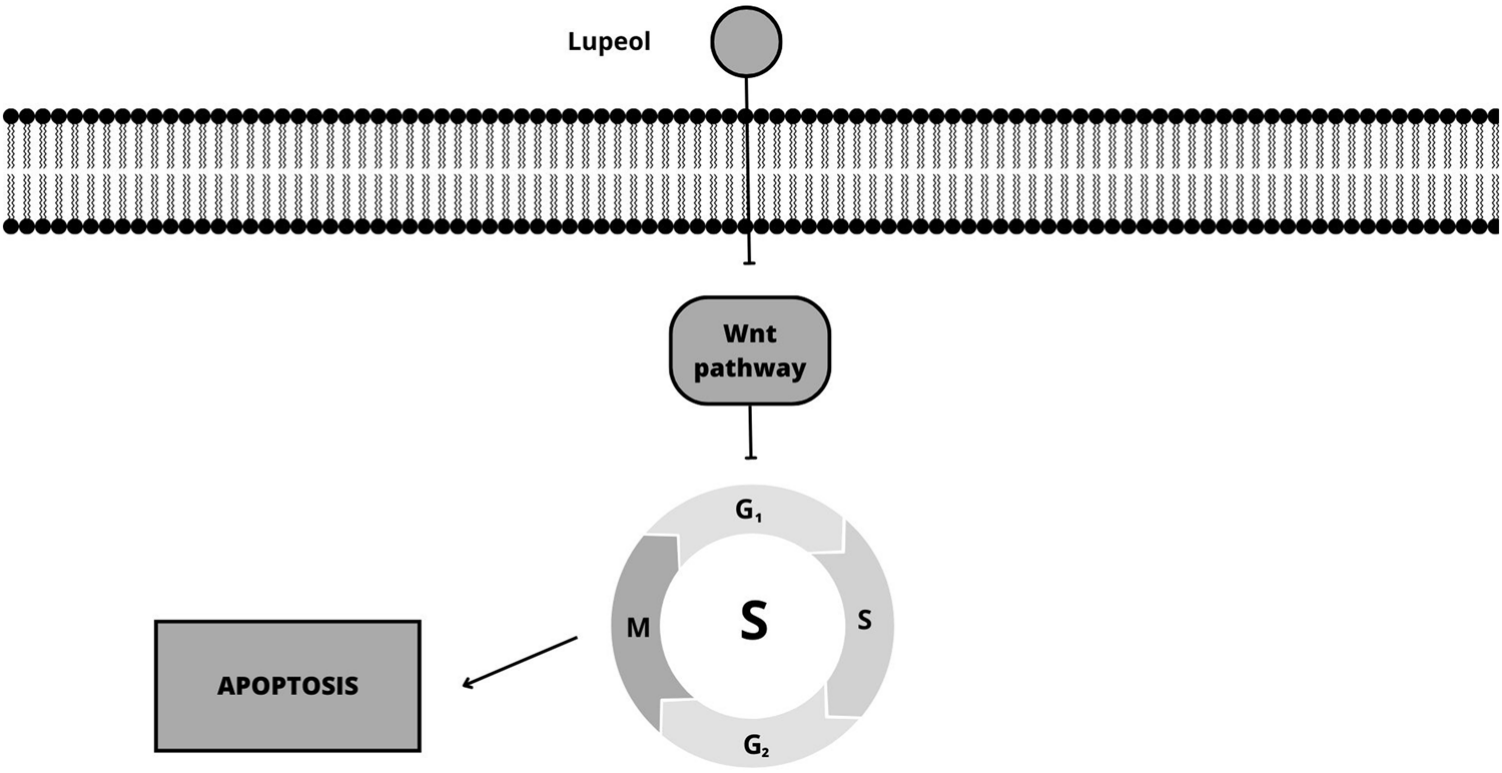
Schematic mechanisms involved in the effect of lupeol on melanoma cells (Canva for Windows)

Terpenes inhibit cell cycle progression in melanoma by targeting different regulators. For instance, taxol stabilizes microtubules and blocks cell division, inhibiting cell cycle progression [67]. Carvacrol induces cell cycle arrest at the G0/G1 phase by downregulating cyclin D1 and CDK4/6 [68]. Artesunate exhibits its antitumor activity in uveal melanoma cells by inhibiting the accumulation of β-catenin and activating specific downstream genes, including c-Myc and CDK1 (Fig. 4), leading to the suppression of cancer cell growth and proliferation [69]. Lupeol demonstrated the ability to suppress the growth of melanoma cells (Mel-928, Mel-1241, Mel-1011) by interfering with the Wnt (Wingless signaling)/β-catenin pathway. It achieves this by effectively blocking the Wnt signaling pathway, a crucial pathway involved in cell proliferation and survival (Fig. 5). This inhibition of the Wnt/β-catenin pathway contributes to the antitumor effects of lupeol on melanoma cells [70].

Terpenes modulate the expression of several genes involved in the regulation of cell growth and survival, including the tumor suppressor gene p53 [71]. Terpenes exhibit anti-angiogenic effects by suppressing the expression of pro-angiogenic factors, such as VEGF (vascular endothelial growth factor) and bFGF (basic fibroblast growth factor). Inhibition of angiogenesis is critical for preventing tumor growth and metastasis [35, 66].

Terpenes inhibit angiogenesis in melanoma cells through various molecular pathways. For example, limonene downregulates VEGF and MMP-9 (matrix metallopeptidase 9) expression [72], but betulinic acid suppresses the expression of VEGF and MMP-2 (matrix metallopeptidase 2) [73], whereas thymoquinone downregulates HIF-1α (Hypoxia-inducible factor 1-alpha**)** and VEGF [74].

Terpenes can modulate chronic inflammation in melanoma cells by targeting different inflammatory pathways. For instance, α-bisabolol inhibits the expression of TNF-α and IL-1β [75]. Furthermore, terpenes such as β-elemene, perillyl alcohol, and limonene inhibit melanoma cell proliferation and induce apoptosis [76–78]. They can also inhibit melanoma cell migration and invasion by regulating the expression of matrix metalloproteinases (MMPs) and tissue inhibitors of metalloproteinases (TIMPs) [79–81]. Additionally, certain terpenes can enhance the antitumor activity of conventional chemotherapeutic agents, such as doxorubicin, cisplatin, and temozolomide, through various mechanisms [82–84].

## Advantages and limitations of using terpenes as anticancer agents

Terpenes have gained increasing attention as potential anticancer agents due to their various pharmacological properties, including their ability to induce apoptosis, inhibit proliferation, sensitize cancer cells to chemotherapy drugs, and overcome resistance to targeted therapies. Furthermore, terpenes are widely distributed in plants and are easy to extract, making them a relatively inexpensive source of potential anticancer agents.

However, the use of terpenes as anticancer agents also has some limitations. One of the major limitations is their low bioavailability, which can limit their efficacy in vivo [85]. Terpenes are highly hydrophobic molecules, poorly soluble in water, and difficult to absorb and distribute in the body. Several strategies have been proposed to enhance the bioavailability of terpenes, such as encapsulation in liposomes or cyclodextrins [86, 87].

Another limitation of terpenes in anticancer therapy is their potential toxicity. Although terpenes are generally considered safe, some terpenes can exhibit cytotoxic effects on normal cells (i.e., eugenol at high concentrations) [88]. Therefore, it is important to carefully evaluate the safety and toxicity of terpenes before their clinical use.

Finally, the regulatory status of terpenes as drugs can also pose a challenge to their development as anticancer agents. Terpenes are classified as natural products and are subject to less stringent regulations than synthetic drugs. However, this can also limit their commercial potential due to the lack of intellectual property protection and the challenges in obtaining regulatory approval [89].

## Clinical studies on the use of terpenes for melanoma treatment

Quite recently, some clinical studies have investigated the efficacy of terpenes in the treatment of melanoma. For instance, perillyl alcohol and limonene were studied in phase II of clinical trials, when evaluating their safety and efficacy in patients with advanced melanoma. Of note, both terpenes (perillyl alcohol and limonene) were well-tolerated, with no dose-limiting toxicities observed but, no objective responses were observed, with a median time to progression of 2 months [46, 90]. Similarly, a phase I clinical trial revealed that thymoquinone was well-tolerated, with no dose-limiting toxicities observed. However, no objective responses were observed, and the median time to progression was 2 months [91].

Terpenes may have potential as adjuvants to standard treatments, such as chemotherapy and immunotherapy.

## Potential use of terpenes in combination with other anticancer therapies

Combination therapy, using two or more agents with different mechanisms of action, has become an important strategy in cancer treatment. In recent years, there has been increasing interest in using terpenes in combination with other anticancer therapies to enhance their efficacy and overcome drug resistance. For example, β-caryophyllene enhanced the antitumor activity of doxorubicin in melanoma cells [92]. Similarly, α-humulene enhanced the antitumor activity of cisplatin in melanoma cells [93]. Linalool potentiated the antitumor activity of temozolomide in melanoma cells [94].

Immunotherapy, such as immune checkpoint inhibitors, has revolutionized the treatment of melanoma. However, not all patients respond to immunotherapy and there is a need to improve its efficacy. Terpenes have been shown to have immunomodulatory effects and may enhance the efficacy of immunotherapy. For example, β-caryophyllene enhanced the antitumor activity of anti-PD-1 immunotherapy in a mouse model of melanoma [95]. Similarly, β-elemene enhanced the antitumor activity of anti-PD-1 immunotherapy in a mouse model of melanoma by increasing T-cell infiltration and activation [96].

Radiation therapy is used in the treatment of melanoma, but its efficacy is limited by radiation resistance. Terpenes enhanced the radiosensitivity of cancer cells, potentially improving the efficacy of radiation therapy. For example, β-elemene enhanced the radiosensitivity of melanoma cells by inducing cell cycle arrest and apoptosis [97]. Similarly, thymoquinone enhanced the radiosensitivity of melanoma cells by inducing apoptosis and inhibiting DNA repair [98].

Targeted therapies, including BRAF and MEK inhibitors, have shown promise in the treatment of melanoma. However, resistance to these therapies is a major clinical problem. It has been shown that terpenes exert synergistic effects with targeted therapies, potentially overcoming resistance. For example, β-caryophyllene enhanced the antitumor activity of vemurafenib, a BRAF inhibitor, in melanoma cells [99]. Similarly, α-humulene enhanced the antitumor activity of trametinib, a MEK inhibitor, in melanoma cells [100].

## Future directions for research on terpenes and melanoma treatment

There is growing interest in the potential use of terpenes for the treatment of melanoma, and future research in this area is likely to focus on several key areas. In in vivo melanoma models, terpenoids have shown the ability to increase the median overall survival time of animals with tumors, reduce tumor volume, decrease the expression of metastasis-associated chemokines and receptors, as well as lymph node metastasis, decrease the number and size of metastatic foci, alter the tumor microenvironment and the surrounding adipose tissue of lymph nodes and inhibit angiogenesis. Notably, plant-derived terpenoids generally exhibit lower-to-no toxicity towards non-cancerous cells or even enhance their photoprotection [57]. Further preclinical and clinical studies are needed to fully evaluate the safety and efficacy of terpenes in combination with other therapies for the treatment of melanoma. Although early studies have shown promising results, more extensive research is needed to establish the optimal doses, treatment regimens, and potential side effects of terpene-based therapies [98, 101–103]. One key advantage they possess over traditional chemotherapeutic agents is their lower cytotoxicity. Research conducted over the past eight years has revealed several effects of plant terpenoids on in vitro melanoma models. These include: demonstrating dose-dependent cytotoxicity, inducing apoptosis, necrosis, or autophagy, triggering the increased generation of reactive oxygen species, oxidative stress, and disruption of mitochondrial membrane potential, reducing oxygen consumption rate, extracellular acidification rate, oxidative phosphorylation, and the maximal respiratory capacity of the electron transport system, inducing endoplasmic reticulum stress, causing cell cycle arrest, inducing DNA damage, decreasing the expression and activity of proteins involved in melanogenesis, interfering with cell signaling pathways responsible for cell growth, proliferation, migration, adhesion, and invasion, reducing the expression of angiogenesis-related cytokines, inhibiting epithelial-mesenchymal transition, exhibiting radio- and photosensitization properties and displaying synergistic effects with other natural compounds or chemotherapeutics [57].

Despite the numbers of experiments, there is a need to explore the mechanisms underlying the effects of each terpene on melanoma cells. Further research in this area could provide valuable insights into the potential therapeutic applications of terpenes [98, 101–103]. There is a need to investigate the potential use of terpenes as adjuvant therapies in combination with immunotherapy. Terpenes may modulate immune responses and may therefore have the potential to enhance the effectiveness of immunotherapy for melanoma [104, 105]. Additionally, there is a need to explore the potential use of terpenes as chemopreventive agents for melanoma. Future research could investigate the potential use of these compounds for the prevention of melanoma [25, 106].

## Conclusions

In recent years, there has been increasing interest in using terpenes in combination with other anticancer therapies to enhance their efficacy and overcome drug resistance. In combination with chemotherapy, β-caryophyllene, α-humulene, and linalool enhance the efficacy of chemotherapy agents in melanoma cells. Terpenes due to their immunomodulatory effects may enhance the efficacy of immunotherapy, especially, β-caryophyllene and β-elemene, which enhanced the antitumor activity of anti-PD-1 immunotherapy in mouse models of melanoma. In combination with radiation therapy, β-elemene, and thymoquinone may enhance the radiosensitivity of melanoma cells. Given the efficacy of terpenoids, future research must focus on conducting thorough preclinical evaluations of toxicity, bioavailability, pharmacodynamics, biomarkers, and comprehensive investigations into tumor suppression. Future studies are likely to focus on exploring the optimal use of terpenes in combination with other therapies, investigating the underlying mechanisms of their effects on melanoma cells, and exploring their potential use as adjuvant therapies or chemopreventive agents.

## Data Availability

Not applicable.

## Abbreviations

bFGF: Basic fibroblast growth factor
BRAF: V-raf murine sarcoma viral oncogene homolog B1
HDAC: Histone deacetylase activity
HIF-1α: Hypoxia-inducible factor 1-alpha
HPGD: 15-Hydroxyprostaglandin dehydrogenase
IL-1β: Interleukin 1 beta
IL-6: Interleukin 6
MAPK/ERK: Mitogen-activated protein kinase (MAPK)/Extracellular signal-Regulated Kinase (ERK)
MEK: Mitogen-activated protein kinase kinase
MMP-9: Matrix metallopeptidase 9
PI3K/AKT/mTOR: Phosphatidylinositol 3-kinase (PI3K)/protein kinase B (AKT)/mammalian target of the rapamycin (mTOR)
TIMPs: Tissue inhibitors of metalloproteinases
TNF-α: Tumor necrosis factor α
VEGF: Vascular endothelial growth factor
Wnt: Wingless signaling
